# Is Bladder Tumor Location Associated with Prostate Cancer Detection after Intravesical Bacillus Calmette-Guérin Instillation?

**DOI:** 10.1371/journal.pone.0103791

**Published:** 2014-07-29

**Authors:** Sungwoo Hong, Seong-Cheol Kim, Taekmin Kwon, In Gab Jeong, Choung-Soo Kim, Hanjong Ahn, Jun Hyuk Hong

**Affiliations:** 1 Department of Urology, University of Ulsan College of Medicine, Asan Medical Center, Seoul, Korea; 2 Department of Urology, Dankook University College of Medicine, Cheonan, Korea; 3 Department of Urology, Inje University Medical College, Pusan, Korea; National Health Research Institutes, Taiwan

## Abstract

**Objectives:**

The aim of this study was to evaluate the effect of bladder tumor (BT) location on prostate cancer (PCa) detection in patients with elevated PSA levels after intravesical BCG instillation.

**Methods:**

Between February 2004 and January 2013 prostate biopsies were performed in 59 non-muscle invasive bladder cancer (NMIBC) patients whose PSA level were elevated (≥3 ng/ml) after a 6 week course of intravesical BCG (Oncotice, 12.5 mg in 50 ml normal saline). Differences in PCa detection according to the BT location [bladder neck and/or trigone (Group 1, n = 22) vs. other locations (Group 2, n = 37)] were evaluated. The Fisher's exact test and the Mann-Whitney U test were used to evaluate the association between categorical and continuous variables, respectively.

**Results:**

A total of 14 patients (23.7%) were diagnosed with PCa. The mean ± standard deviation (SD) PSA before intravesical BCG instillation and prostate biopsy were 1.36±1.04 ng/ml in Group 1 and 1.09±1.12 ng/ml in Group 2 (*P* = 0.633), and 6.05±3.57 ng/ml in Group 1 and 5.13±3.88 ng/ml in Group 2 (*P* = 0.378), respectively. Interestingly, whereas PCa was detected upon biopsy in only one patient in Group 1 (4.5%), 13 cases were detected in Group 2 (35.1%) (*P* = 0.009).

**Conclusions:**

PCa detection after intravesical BCG was highly associated with BT location. Prostate biopsy should therefore be considered when PSA level is elevated after BCG instillation and his BT is located far from the bladder neck.

## Introduction

Approximately 92% of all new cases of urothelial bladder cancer were classified as non-muscle-invasive bladder cancer (NMIBC) in a recent population-based study [1], a higher figure than those cited in earlier reports [2], [3]. NMIBC includes Ta (noninvasive papillary carcinoma) and T1 (invasion of the lamina propria) tumors as well as carcinoma *in situ*, which account for 70, 20, and 10% of NMIBCs, respectively [4]. Since the first report of treatment with intravesical bacillus Calmette-Guérin (BCG) instillations by Morales et al. [5], BCG has been established as the standard of care after transurethral resection of high-risk NMIBC.

Although the majority of the patients tolerate the intravesical BCG instillations well, a number of side effects have been reported [6]–[8]. Adverse events are generally due to intravasation of live bacteria. Although clinically symptomatic granulomatous prostatitis (GP) reportedly affects 10% of patients after intravesical BCG instillation [9], it has been shown that levels of serum prostate-specific antigen (PSA) increase in up to 40% of BCG-treated patients, and return to baseline by 3–12 months post-treatment [10], [11]. Therefore, Beltrami et al. reported that biopsy did not appear mandatory in patients with PSA increase after BCG instillation, even if a positive digital rectal exam (DRE) was detected [10].

The exact mechanism by which intravesical BCG instillation causes GP is not understood. It may be a result of reflux of urine containing BCG into the prostatic ducts. Therefore, serum PSA elevations after intravesical BCG instillation can be interpreted differently in patients with bladder tumors located at or near the bladder neck from the patients with BTs located away from the bladder neck. To our knowledge, the effect of BT location on eventual prostate cancer (PCa) detection in NMIBC patients with elevated serum PSA following intravesical BCG instillation has not previously been documented. The aim of this study was to evaluate whether there are statistically and clinically significant associations between BT location and the occurrence of PCa after intravesical BCG instillation.

## Patients and Methods

### 2.1 Patient Population

This retrospective study enrolled 59 patients with NMIBC who underwent prostate biopsy for suspected PCa after intravesical BCG instillation at our institution between February 2004 and January 2013. Demographic information, dates of BCG instillations, dates of prostate biopsies, baseline laboratory findings, pathologic results from transurethral resection of BT (TURBT) or radical prostatectomy (RP), and descriptions of operative findings were taken directly from patients' medical records. Indications for intravesical BCG instillation included multiple tumors, a tumor with lamina propria invasion, carcinoma *in situ*, and tumor recurrence.

Men who were younger than 50 years, had a documented history of prostate biopsy before the occurrence of BT, PSA >3 ng/ml before TURBT, received 5-alpha reductase inhibitors, or required repeat urinary tract manipulation, such as urethral dilation or intermittent catheterization due to stricture of the urethra, were excluded from analysis. Also, patients who could not complete a 6 week course of BCG instillation due to BCG sepsis or BCG-induced cystitis were excluded.

Study participants were divided into two groups according to the BT location: those who had BT lesions around the bladder neck (Group 1), and those whose tumors were not located near the bladder neck (Group 2). The size of the BT lesion was determined from computed tomography or cystoscopic findings. Patients with multiple BT lesions were grouped according to the location of the largest main mass. The Institutional Review Board of Asan Medical Center approved this study and exempted it from the informed consent requirement because we only retrospectively accessed a de-identified database for this study

### 2.2 BCG Protocols

All patients received a 6 week course of Connaught strain BCG intravesically (Oncotice, 12.5 mg in a volume of 50 ml normal saline) 3 weeks after TURBT, followed by cystoscopy 4 weeks after the final instillation. Patients were instructed to limit fluid intake for 8–12 hours before the treatment, and to have no fluid intake for 4 hours before treatment. Patients were instructed to refrain from voiding during the initial 2 hours after instillation and to avoid direct skin contact during and after urinating, as it may cause skin rash and irritation. Lastly, patients were encouraged to increase fluid intake, to flush the remaining BCG from the urinary bladder thoroughly. Intravesical BCG instillation was started a median of 21 days (range, 17–28) after TURBT.

### 2.3 Prostate Biopsy Protocol and Pathologic Specimens

Blood samples for serum PSA determination were performed in all patients before TURBT and at the end of the intravesical BCG instillation and before follow up cystoscopy. A digital rectal exam was always conducted before checking serum PSA. A prostate biopsy was performed when serum PSA was greater than 3 ng/ml or an abnormal nodule was palpated on the DRE. Twelve core biopsies were routinely obtained under transrectal ultrasonography guidance by three experienced radiologists. The prostate was biopsied bilaterally at the base, midgland, and apex, with at least six biopsies per side. Additional cores were taken at the discretion of the radiologists for suspicious abnormalities such as hypoechoic lesions. The TNM classification (American Joint Committee on Cancer, 7^th^ edition, 2010) was used for pathologic staging, and the 1973 World Health Organization classification was used for pathologic grading by an experienced uropathologist.

### 2.4 Statistical Analysis

Patients were grouped based on BT location: the bladder neck (± trigone) vs. all other BT locations. Fisher's exact test was used to evaluate associations between categorical variables. Differences in variables with a continuous distribution across categories were assessed using the Mann-Whitney U test. All reported p-values are two-sided, and statistical significance was set at p<0.05. Statistical tests were performed with SPSS v.18.0 (SPSS, IBM Corp., Armonk, NY, USA).

## Results

The baseline demographic characteristics of the 59 study participants according to BT location are listed in Table 1. The mean age of Group 1 patients was 69.4 years (range: 56–78 years) and that of Group 2 was 67.5 years (range: 53–78 years). Of the 59 study participants with BT, while GP was found in 18 patients (81.8%) in group 1 and in 19 patients (51.4%) in group 2, PCa was found in 1 patient (4.5%) in group 1 and in 13 patients (35.1%) in group 2 (*P*<.009). A total of 14 patients (23.7% of the study population) were ultimately diagnosed with PCa.

**Table 1:** Patient Characteristics of According to the Bladder Tumor Location.

Variables		Bladder tumor location		*p* value
	Overall (n = 59)	Group 1 (n = 22, 37.3%)	Group 2 (n = 37, 62.7%)	
Mean age at biopsy ± SD, yr	68.2±7.7	69.4±8.4	67.5±6.2	0.132
Median	69.0	65.0	70.0	
Range	53–78	56–78	53–78	
Positive DRE before prostate biopsy, No. (%)	16 (27.1)	4 (18.2)	12 (32.4)	0.404
Mean prostate volume ± SD, ml	37.7±21.6	44.4±29.8	31.4±12.9	0.135
Median	29.0	27.0	27.5	
Range	17.2–115.0	18.4–115.0	17.2–70.0	
Mean PSA before BCG instillation ± SD, ng/ml	1.24±1.09	1.36±1.04	1.09±1.12	0.633
Median	1.30	1.40	1.1	
Range	0.23–2.90	0.46–2.90	0.23–2.80	
Mean PSAD before BCG instillation ± SD, ng/ml/ml	0.04±0.04	0.03±0.04	0.05±0.04	0.154
Median	0.05	0.04	0.06	
Range	0.01–0.15	0.01–0.13	0.01–0.15	
Mean PSA before prostate biopsy ± SD, ng/ml	5.56±3.77	6.05±3.57	5.13±3.88	0.378
Median	4.60	5.02	4.14	
Range	1.44–23.02	1.44–18.63	0.25–23.02	
Mean PSAD before prostate biopsy ± SD, ng/ml/ml	0.19±0.11	0.18±0.11	0.19±0.11	0.917
Median	0.16	0.16	0.16	
Range	0.01–0.54	0.05–0.48	0.01–0.54	
Mean % fPSA before prostate biopsy ± SD, ng/ml	16.12±8.02	16.41±6.20	15.72±4.10	0.746
Median	15.15	15.20	14.80	
Range	8.12–23.84	9.21–22.70	8.12–23.84	
Mean PSAV before prostate biopsy ± SD, ng/ml/yr	4.20±7.41	5.62±10.11	3.21±5.00	0.267
Median	1.61	2.15	1.51	
Range	0.01–42.54	0.21–42.54	0.01–24.34	
Mean No. of previous TUR ± SD	1.8±1.0	2.1±1.0	1.6±1.0	0.050
Bladder tumor stage (%)				0.380
Ta	36 (61.0)	16 (72.7)	20 (54.1)	
T1	19 (32.2)	6 (27.3)	13 (35.1)	
Carcinoma in situ [Concomitant]	4 [5] (6.8)	0 [1] (0)	4 [4] (10.8)	
Bladder tumor grade (%)				1.000
G2	35 (59.3)	14 (63.6)	21 (56.8)	
G3	20 (33.9)	8 (36.4)	12 (32.4)	
Carcinoma in situ [Concomitant]	4 [5] (6.8)	0 [1] (0)	4 [4] (10.8)	
Mean bladder tumor size ± SD, cm	2.0±0.8	2.0±0.8	2.1±0.8	0.885
Multiplicity (%)				0.757
Single	28 (47.5)	10 (45.5)	18 (48.6)	
Multiple	31 (52.5)	12 (54.5)	19 (51.4)	
Prostate biopsy pathology (%)				0.009
PCa (± GP)	14 (23.7)	1 (4.5)	13 (35.1)	
GP (± CI)	37 (62.7)	18 (81.8)	19 (51.4)	
Normal prostatic tissue	8 (13.6)^a^	3 (13.7)	5 (13.5)†	
Mean interval from BCG instillation to biopsy time ± SD, mo	16.5±11.9	16.3±14.3	16.6±10.4	0.924

Group 1, bladder neck (± trigone); Group 2, other.

DRE, digital rectal exam; PSA, prostate-specific antigen; % fPSA, percentage of free PSA; BCG, bacillus Calmette-Guérin; PSAD, PSA density; BT, bladder tumor; TURBT, transurethral resection of bladder tumor; PCa, prostate cancer; GP, granulomatous prostatitis; CI, chronic inflammation.

† One patient had atypical cells on prostate biopsy.

Previous TURBT and pathologic results from the patients diagnosed with PCa after intravesical BCG instillation are listed in Table 2. Nine patients had undergone TURBT twice and three patients had undergone the third TURBT before intravesical BCG instillation. Most of the PCa patients (13/14) had BT lesions relatively far from bladder neck and trigone (Group 2). The remaining patient had a main tumor lesion at the right lateral wall and had one small lesion at the bladder neck, and was classified as Group 1 based on the main tumor location. Seven patients (50.0%) were diagnosed with PCa within 1 year after intravesical BCG instillation.

**Table 2:** Characteristics of Previous TURBT and BT in Patients Diagnosed with Prostate Cancer after BCG Instillation.

		No. of previous TURBT before BCG	Main BT size, cm	BT location	BT stage & grade	Time from final BCG instillation to prostate biopsy, mo
1	62	2	1.4	BN, Rt. lateral and posterior wall	TaG2	2.6
2	54	2	3.3	Dome and Rt. lateral wall	CIS	5.1
3	74	2	2.5	Lt. lateral wall	T1G3 (CIS)	5.8
4	60	2	1.6	Anterior and both lateral wall	TaG2	9.2
5	71	2	2.5	Rt. lateral and posterior wall	T1aG3 (CIS)	10.0
6	78	1 (NUx)	2.2	Dome and Lt.lateral wall	T1G2	10.6
7	70	3	1.1	Rt. lateral wall	TaG2	12.0
8	78	3	2.4	Rt. lateral wall	TaG2	14.9
9	66	2	1.0	Dome	TaG2	23.5
10	74	2	3.0	Both lateral wall	CIS	28.8
11	78	2	1.0	Dome	T1G2	32.7
12	70	3	1.4	Multiple (main; posterior wall)	T1aG3	37.4
13	64	2	3.0	Rt. posterolateral	TaG2	37.9
14	72	1 (NUx)	2.5	Rt. lateral and posterior wall	TaG2	41.9

BT, bladder tumor; TURBT, transurethral resection of bladder tumor; BCG, bacillus Calmette-Guérin; BN, bladder neck; CIS, carcinoma in situ.

Table 3 lists serum PSA profiles and pathologic results from biopsy and RP in the PCa patients, and lists the time from the final intravesical BCG instillation to the prostate biopsy. Mean time from BCG instillation to prostate biopsy was 19.4 months (range: 2.6–41.9 months). On prostate biopsy, 7 patients (50.0%) had a Gleason score (GS) of 6, and 7 patients had a GS of 7 or higher (50.0%). Nine patients (64.3%) had undergone RP (three open and six robot-assisted).

**Table 3:** Serum PSA Profiles and Pathologic Results of Biopsy and Radical Prostatectomy in Prostate Cancer Patients According to the Time from BCG to Prostate Biopsy.

	N	Age	Time from BCG to prostate biopsy, mo	Before BCG instillation			Before prostate biopsy				Positive core	Biopsy GS	RP pathology
					PSA	PSAD		PSA	PSAV	PSAD			
				DRE	ng/ml	ng/ml/ml	DRE	ng/ml	ng/ml/yr	ng/ml/ml			
Group A	1	62	2.6	−	2.90	0.04	+	9.20	29.1	0.14	1/12	GS 7 (3+4) (GP−)	T2aGS6,GP(+)
	2	54	5.1	−	2.00	0.05	+	4.00	4.71	0.10	3/12	GS 6 (3+3) (GP+)	T2cGS6,GP(+)
	3	74	5.8	−	1.90	0.06	−	3.80	3.93	0.11	2/12	GS 7 (4+3) (GP+)	T2cGS7(3+4),GP(+)
	4	60	9.2	−	2.90	0.11	−	7.70	6.26	0.30	6/12	GS 7 (4+3) (GP−)	T3aGP(+)†
	5	71	10.0	−	1.50	0.11	−	3.50	2.40	0.25	2/12	GS 6 (3+3) (GP+)	ADT
	6	78	10.6	−	2.60	0.10	+	4.90	2.60	0.19	4/12	GS 8 (4+4) (GP−)	ADT
	7	70	12.0	−	2.60	0.12	+	11.30	8.49	0.54	6/12	GS 8 (4+4) (GP−)	RT+ADT
Group B	8	78	14.9	−	1.60	0.05	−	7.00	4.35	0.24	1/12	GS 6 (3+3) (GP+)	RT
	9	66	23.5	−	2.10	0.11	−	4.30	1.12	0.22	1/12	GS 6 (3+3) (GP−)	T2cGS6,GP(+)
	10	74	28.8	−	2.30	0.11	+	4.60	0.96	0.22	1/12	GS 6 (3+3) (GP−)	T2cGS8,GP(−)
	11	78	32.7	−	2.80	0.08	−	9.70	2.53	0.29	1/12	GS 7 (3+4) (GP−)	ADT
	12	70	37.4	−	2.60	0.10	+	3.50	0.29	0.13	1/12	GS 6 (3+3) (GP+)	T2aGS6,GP(+)
	13	64	37.9	−	2.00	0.11	−	5.20	1.17	0.30	3/12	GS 7 (3+4) (CGP−)	T2cGS7(3+4),GP(+)
	14	72	41.9	−	2.20	0.05	+	3.40	0.34	0.08	1/12	GS 6 (3+3) (CG+)	T2aGS6,CG(+)

Group A, less than 1 yr from BCG to biopsy; Group B, more than 1 yr from BCG to biopsy.

† Could not be evaluated due to neoadjuvant HT.

PSA, prostate-specific antigen; BCG, bacillus Calmette-Guérin; GS, Gleason score; GP, granulomatous prostatitis; DRE, digital rectal exam; PSAD, PSA density; PSAV, PSA velocity; ADT, androgen deprivation therapy; RT, radiotherapy.

## Discussion

Leibovici et al. [11] reported that serum PSA elevation peaks after the third or fourth intravesical BCG instillation, and elevated serum PSA may persist for up to 3 months after treatment. Beltrami et al. [10] reported a progressive increase in serum PSA during early intravesical BCG instillations and a return to basal levels after several months. In the present study, 53 patients (89.8%) experienced an increase in serum PSA of 0.1–18.1 ng/ml after intravesical BCG instillation. Establishing an association between intravesical BCG instillation and serum PSA elevation would obviate the need for a prostate biopsy, decreasing the risks and adverse effects of prostate biopsy in this patient population.

The prevalence of GP after BCG therapy has been reported to be 1.3–40% [12], [13]. Non-specific GP is usually an incidental finding, with an incidence of 3.4% in an unselected series of patients [14]; it is also detected in 0.44% of routine RP specimens [15]. GP is even detected in 0.29–3.3% of prostate biopsies [15], [16]. Therefore, considering the prevalence of GP in patients who receive intravesical BCG, the inflammatory process cannot be distinguished from PCa in cases of serum PSA elevation. Of all the participants, only GP was detected in 37 patients (62.7%) on prostate biopsy. Of them, 21 patients (56.8%) underwent prostate biopsy within 1 year of BCG instillation, and the remaining patients (43.2%) underwent biopsy more than a year after BCG therapy. Of the PCa patients without GP on prostate biopsy, five underwent RP, and all but one had GP in the prostatectomy specimen. Therefore, BT location could be used to interpret elevations in serum PSA after intravesical BCG instillation. This is supported by the observation that the patients who had lesions adjacent to the bladder neck had a higher incidence of GP (72.7%) than those who did not (60.0%).

Of the PCa patients, seven were diagnosed within 1 year after the initiation of intravesical BCG instillation. As mentioned earlier, more than 90% of all new cases of malignant BT are classified as NMIBC. Due to the increased use of BCG in NMIBC, it is important to understand the kinetics of serum PSA elevation after intravesical BCG instillation precisely. When patients diagnosed with PCa within 1 year after intravesical BCG instillation are compared to those who diagnosed more than a year later, two convincing hypotheses emerge. It is possible that the detection of pre-existing PCa is facilitated by the rapidly increased serum PSA caused by intravesical BCG instillation. The other possibility is that BCG instillation enhances *de novo* PCa carcinogenesis by increasing inflammatory processes.

To our knowledge, no studies have been conducted on PCa detection according to BT location in NMIBC patients who underwent intravesical BCG instillation. Although our study population is relatively small, our results indicate that PCa detection after intravesical BCG instillation might be associated with BT location on TURBT. Previous studies have only revealed a correlation between serum PSA elevation and intravesical BCG instillation. In our study, the BT lesions in 13 PCa patients (92.9%) were located away from the bladder neck. It is likely that greater amounts of BCG were absorbed into the prostate tissue in patients with BT lesions adjacent to the bladder neck, stimulating the release of serum PSA. In 2003, Lopez et al. [17] reported that raised serum PSA was observed in 87.5% of 24 cases during intravesical BCG instillation, although the increase was only significant in patients who had undergone transurethral resection of the prostate (TURP). They concluded that intravesical BCG instillation produced an increase in serum PSA levels and this variation was higher in patients with history of TURP. Based on that theory, BT patients with lesions relatively far from the bladder neck might have a greater chance of being diagnosed with PCa if their serum PSA is elevated following BCG therapy. In the present study, only one patient ultimately diagnosed with PCa had a BT lesion at the bladder neck, but the main tumor mass in this patient was a 2 cm papillary mass located at right lateral wall. Although a Cox regression analysis to evaluate significant factors predicting PCa detection in NMIBC patients who received intravesical BCG instillation was not conducted due to the small study cohort, our findings suggest that there may be a relationship between BT location and serum PSA elevation.

Chronic prostatic inflammation, a common condition in humans, can be initiated by several stimuli that induce a proinflammatory state in the prostatic microenvironment. Chronic inflammation (due to exposure to infectious agents and/or environmental factors) is involved in the pathogenesis of about 20% of human cancers, including those of the stomach, liver, and large intestine [18], [19]. Epidemiologic, histopathologic, and molecular pathologic studies provide emerging evidence of the possible role of prostatic inflammation in PCa pathogenesis and progression [20]. Similarly, PCa evolves over a long period of time through precancerous modifications, eventually progressing to a clinically significant PCa. Mukamel et al. reported that GP may simulate PCa [21], and PCa has been reported in 10–14% of patients with clinically diagnosed GP [22], [23]. It may therefore be appropriate to wait several months after BCG installation before conducting a prostate biopsy to confirm PCa.

This study has several limitations. First, although the study population was selected carefully, the problems inherent in a retrospective study are unavoidable and can influence the results. Secondly, although the present study has yielded some preliminary findings, the number of our study participants is relatively small. For this reason, these findings cannot be generalized to the broader community based on the present study alone. Thirdly, we did not have enough the period under observation to investigate the relationship between GP and PCa. Lastly, because serial serum PSA determinations were not conducted each time the patients underwent BCG instillation, it is not possible to define the serum PSA changes induced during the 6 week course of BCG.

It is a noteworthy finding that BT location may be associated with PCa development in NMIBC patients after intravesical BCG instillation. Of the PCa patients, seven patients had a GS of 6, but seven patients had a GS of ≥7, which is generally considered unfavorable. Two PCa patients diagnosed within 1 year of BCG installation had a GS of 8. Even though serum PSA levels found to be elevated after intravesical BCG instillations might return to basal levels after several months, the possibility of PCa should not be discounted.

## Conclusions

PCa detection after intravesical BCG instillation might be associated with BT location on TURBT. In BT cases that are located far from the prostate, prostate biopsy should be considered in patients with suspected PCa regardless of the time since intravesical BCG instillation.
